# Supplementary material for: Gene editing and mutagenesis reveal inter-cultivar differences and additivity in the contribution of TaGW2 homoeologues to grain size and weight in wheat
Source: Theor Appl Genet. 2018 Aug 22;131(11):2463–75. doi: 10.1007/s00122-018-3166-7 (PMC6208945; doi:10.1007/s00122-018-3166-7)
Supplement: Supplementary file 1 — Supplementary material 1 (DOCX 451 kb) [file 122_2018_3166_MOESM1_ESM.docx]

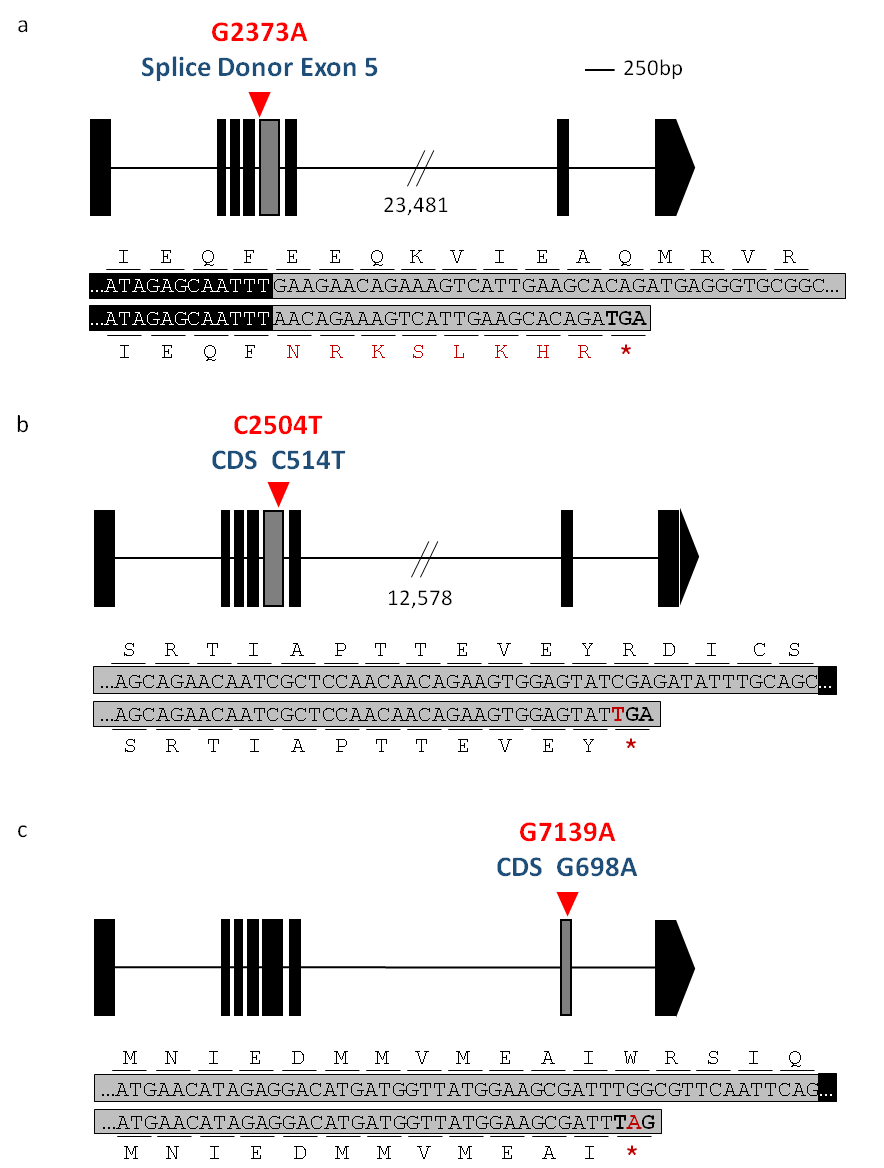


**Figure S1. A schematic of the position of the EMS-induced mutations on the *TaGW2* gene A, B and D homoeologues and the predicted change in proteins.**

The *TaGW2* gene structure for the three homoelogues of *TaGW2;* A (a), B (b), and D (c) (RefSeq V1.0). The location of the mutations are indicated with a red triangle and the exact positions for the gDNA and CDS are indicated in red and blue font respectively. The CDS sequence alignment is shown for the region surrounding the mutations for the WT (top) and mutant line (bottom) for each homoeologue. The mutated base and resulting amino acid changes are indicated with red font.


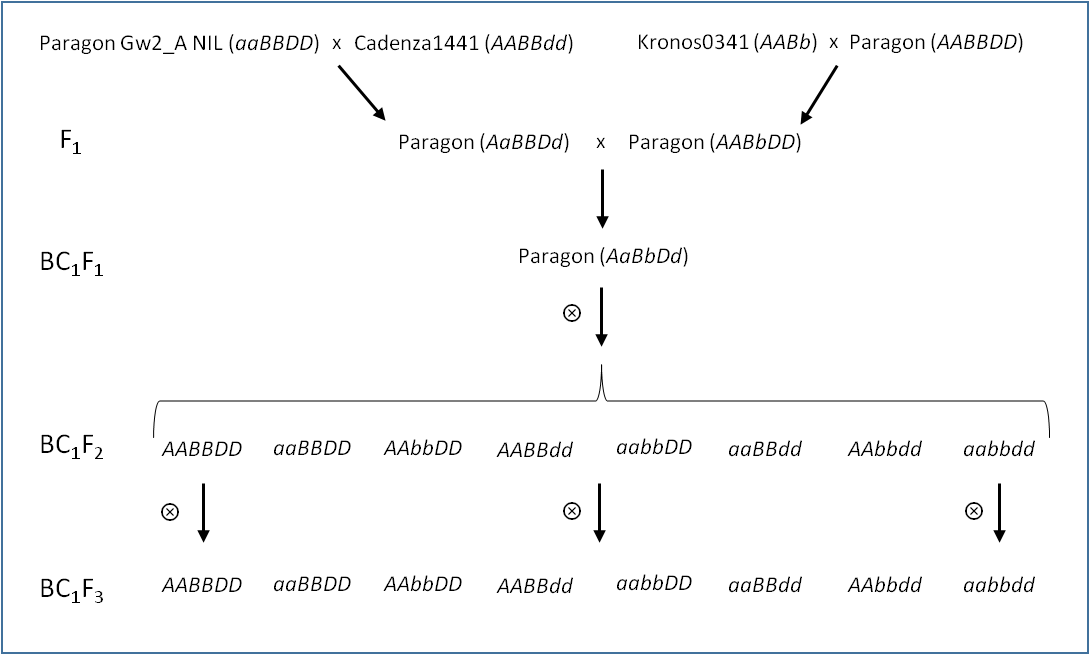


**Figure S2. The workflow chart of *TaGW2* gene mutants development in Paragon**

Crossing scheme used for the introduction of the identified TILLING mutations and development of the BC_1_F_3_ mutant lines. Arrows indicate progression through to the next generation as indicated on the left hand side and small crossed circles indicate self-pollination. Selected alleles are indicated by either upper case letters for wild-type or lower case for mutants.


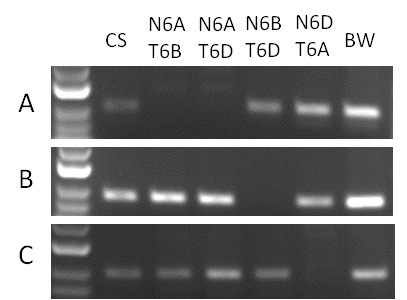


**Figure S3. Validation of the *TaGW2* gene homoeologue-specific primers for RT-PCR**

PCR amplification of cv. Chinese Spring (CS) nullisomic-tetrasomic lines with the genome-specific primer sets: **A)** GW2A_RT_F and GW2A_RT_R, **B)** GW2B_RT_F and GW2B_RT_R, and **C)** GW2D_RT_F and GW2D_RT_R. PCR was performed using DNA isolated from cv. Chinese Spring (CS), four nullisomic-tetrasomic lines (N6A-T6B, N6B-T6D, and N6DT6A), and cv. Bobwhite (BW). From the top to the bottom, the DNA ladder fragment lengths are 400 bp, 300 bp, 200 bp, and 150 bp.


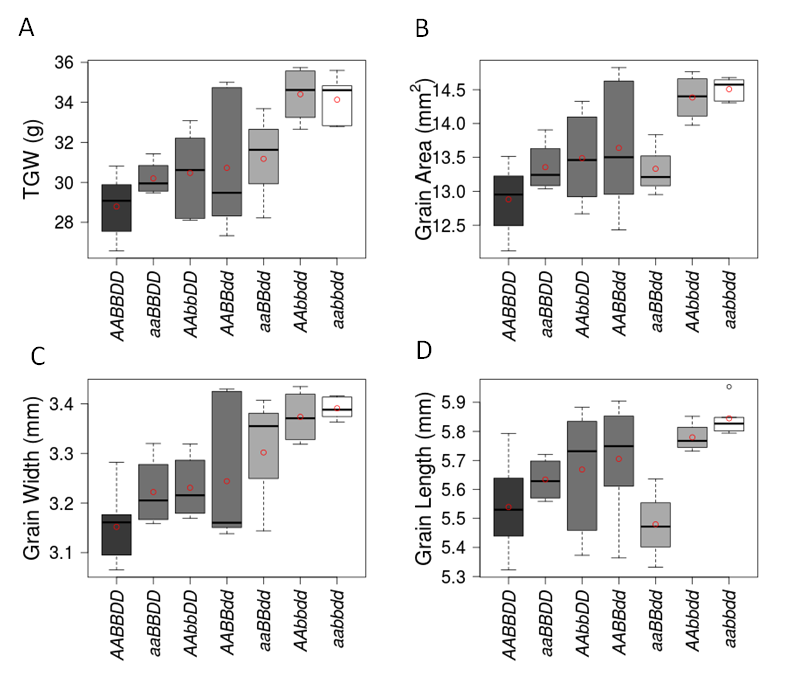


**Figure S4. Dosage-dependent phenotypic effects of the *TaGW2* homoeologue mutants**

The grain morphometric and TGW traits CRISPR-Cas9-induced *TaGW2* mutants carrying single-, double-, and triple-gene KO mutations in cv. Bobwhite were assessed in the preliminary experiment conducted in the spring of 2017. No plants carrying the double-gene KO mutations in the A and B genome homoeologues were obtained in this experiment. Box and whisker plots were used to show the grain morphometric and TGW traits’ distribution. The TGW, grain area, grain width, and grain length trait distributions were shown in **A)**, **B)**, **C)**, and **D),** respectively. The mean value for each genotype was shown as a red circle. The genotypes of the *TaGW2* gene locus are shown on the horizontal axis. The A/a, B/b, and D/d letters in the genotype designation represent the A, B, and D genome homoeologues, respectively, with lower and upper case letters corresponding to the mutated and wild-type alleles, respectively.


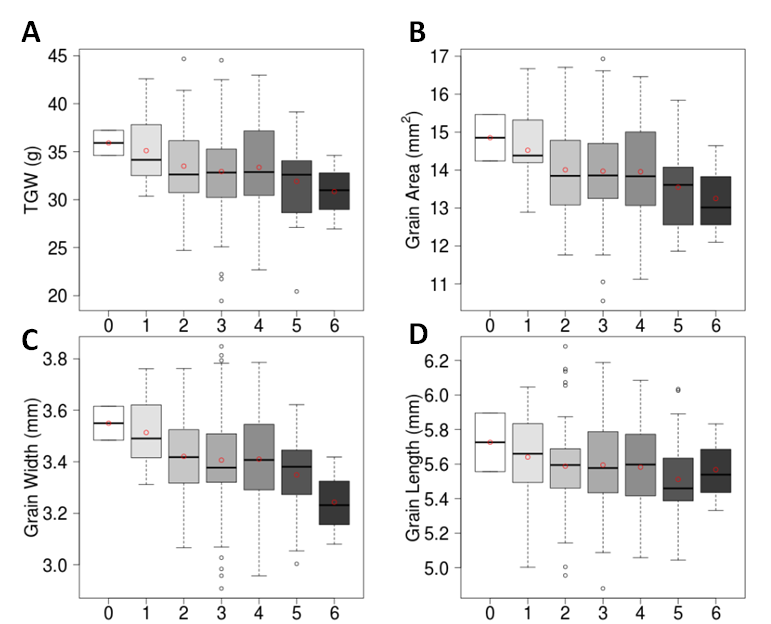


**Figure S5. The grain morphometric and TGW trait distribution in the F2 lines carrying different number of the functional *TaGW2* gene copies.**

The grain morphometric and TGW traits were investigated in the F2 population developed by crossing triple-gene KO mutant of Bobwhite with cv. Thatcher. Box and whisker plots were used to show the trait distribution. The TGW, grain area, grain width, and grain length trait distributions are shown in **A)**, **B)**, **C)**, and **D),** respectively. The mean value for each group of lines carrying the same number of the functional *TaGW2* gene copies is shown as a red circle. The number of functional *TaGW2* gene copies is shown on the horizontal axis.


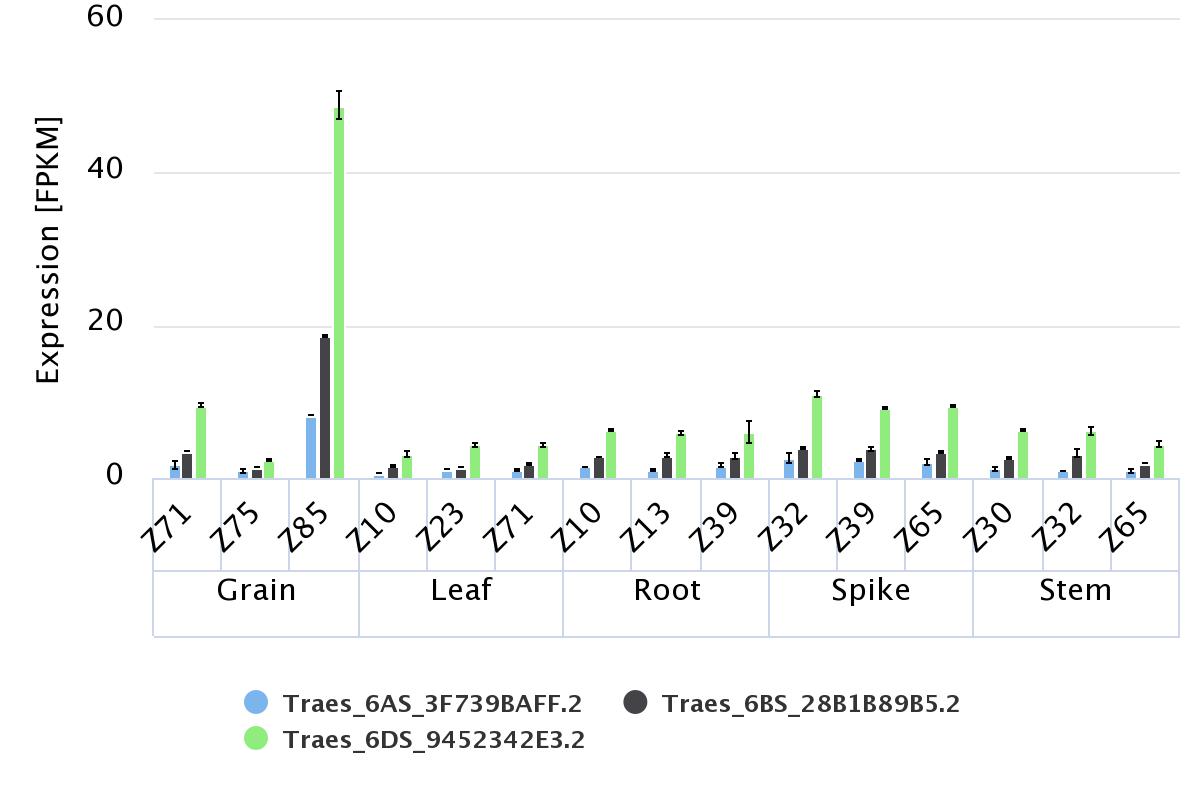


**Figure S6. Expression levels of the *TaGW2-A1*, *-B1* and *–D1* gene homoeologs in different tissues of cv. Chinese Spring.**

The expression values of *TaGW2-A1*, *-B1* and *-D1* were obtained from the WheatEXP website (<https://wheat.pw.usda.gov/WheatExp/>). The results are based on the RNA-Seq data generated by Choulet *et.al.* (2014). Traes_6AS_3F739BAFF.2, Traes_6BS_28B1B89B5.2 and Traes_6DS_9452342E3.2 are gene IDs corresponding to *TaGW2-A1*, *-B1* and *-D1*, respectively. The grown stages are shown according to Zadoks Cereal Development Scale (Zadoks et al. 1974).
