# Supplementary material for: Gene editing and mutagenesis reveal inter-cultivar differences and additivity in the contribution of TaGW2 homoeologues to grain size and weight in wheat
Source: Theor Appl Genet. 2018 Aug 22;131(11):2463–75. doi: 10.1007/s00122-018-3166-7 (PMC6208945; doi:10.1007/s00122-018-3166-7)
Supplement: Supplementary file 2 — Supplementary material 2 (DOCX 63 kb) [file 122_2018_3166_MOESM2_ESM.docx]

**Table S1. The primers for CRISPR/Cas9 target site genotyping.**

**The first round PCR primers**

| Name | Sequence |
| --- | --- |
| GW2T2MiseqF13 | CTCTTTCCCTACACGACGCTCTTCCGATCTCGCTTatggggaacagaataggagg |
| GW2T2MiseqF14 | CTCTTTCCCTACACGACGCTCTTCCGATCTCTAGCatggggaacagaataggagg |
| GW2T2MiseqF15 | CTCTTTCCCTACACGACGCTCTTCCGATCTACAAAatggggaacagaataggagg |
| GW2T2MiseqF16 | CTCTTTCCCTACACGACGCTCTTCCGATCTTTCTCatggggaacagaataggagg |
| GW2T2MiseqF17 | CTCTTTCCCTACACGACGCTCTTCCGATCTAGCCCatggggaacagaataggagg |
| GW2T2MiseqF18 | CTCTTTCCCTACACGACGCTCTTCCGATCTGTATTatggggaacagaataggagg |
| GW2T2MiseqF19 | CTCTTTCCCTACACGACGCTCTTCCGATCTCTGTAatggggaacagaataggagg |
| GW2T2MiseqF20 | CTCTTTCCCTACACGACGCTCTTCCGATCTACCGTatggggaacagaataggagg |
| GW2T2MiseqR21 | CTGGAGTTCAGACGTGTGCTCTTCCGATCTGCTTAaggaagcagatggggcactc |
| GW2T2MiseqR22 | CTGGAGTTCAGACGTGTGCTCTTCCGATCTGGTGTaggaagcagatggggcactc |
| GW2T2MiseqR23 | CTGGAGTTCAGACGTGTGCTCTTCCGATCTAGGATaggaagcagatggggcactc |
| GW2T2MiseqR24 | CTGGAGTTCAGACGTGTGCTCTTCCGATCTATTGAaggaagcagatggggcactc |
| GW2T2MiseqR25 | CTGGAGTTCAGACGTGTGCTCTTCCGATCTCATCTaggaagcagatggggcactc |
| GW2T2MiseqR26 | CTGGAGTTCAGACGTGTGCTCTTCCGATCTCCTACaggaagcagatggggcactc |
| GW2T2MiseqR27 | CTGGAGTTCAGACGTGTGCTCTTCCGATCTGAGGAaggaagcagatggggcactc |
| GW2T2MiseqR28 | CTGGAGTTCAGACGTGTGCTCTTCCGATCTGGAACaggaagcagatggggcactc |
| GW2T2MiseqR29 | CTGGAGTTCAGACGTGTGCTCTTCCGATCTGTCAAaggaagcagatggggcactc |
| GW2T2MiseqR30 | CTGGAGTTCAGACGTGTGCTCTTCCGATCTTAATAaggaagcagatggggcactc |
| GW2T2MiseqR31 | CTGGAGTTCAGACGTGTGCTCTTCCGATCTTACATaggaagcagatggggcactc |
| GW2T2MiseqR32 | CTGGAGTTCAGACGTGTGCTCTTCCGATCTTCGTTaggaagcagatggggcactc |

Note: The target specific primers are shown as lower case letter, the 5 additional barcoding nucleotides are shown as red color upper case letters, the black color upper case letters are part of Illumina Truseq adapter.

**The 96 well plate layout of the first round PCR primers for genotyping of CRISPR/Cas9 induced mutant plants**

|  |  | 1 | 2 | 3 | 4 | 5 | 6 | 7 | 8 | 9 | 10 | 11 | 12 |
| --- | --- | --- | --- | --- | --- | --- | --- | --- | --- | --- | --- | --- | --- |
|  |  | R21 | R22 | R23 | R24 | R25 | R26 | R27 | R28 | R29 | R30 | R31 | R32 |
| A | F20 | F20_R21 | F20_R22 | F20_R23 | F20_R24 | F20_R25 | F20_R26 | F20_R27 | F20_R28 | F20_R29 | F20_R30 | F20_R31 | F20_R32 |
| B | F19 | F19_R21 | F19_R22 | F19_R23 | F19_R24 | F19_R25 | F19_R26 | F19_R27 | F19_R28 | F19_R29 | F19_R30 | F19_R31 | F19_R32 |
| C | F18 | F18_R21 | F18_R22 | F18_R23 | F18_R24 | F18_R25 | F18_R26 | F18_R27 | F18_R28 | F18_R29 | F18_R30 | F18_R31 | F18_R32 |
| D | F17 | F17_R21 | F17_R22 | F17_R23 | F17_R24 | F17_R25 | F17_R26 | F17_R27 | F17_R28 | F17_R29 | F17_R30 | F17_R31 | F17_R32 |
| E | F16 | F16_R21 | F16_R22 | F16_R23 | F16_R24 | F16_R25 | F16_R26 | F16_R27 | F16_R28 | F16_R29 | F16_R30 | F16_R31 | F16_R32 |
| F | F15 | F15_R21 | F15_R22 | F15_R23 | F15_R24 | F15_R25 | F15_R26 | F15_R27 | F15_R28 | F15_R29 | F15_R30 | F15_R31 | F15_R32 |
| G | F14 | F14_R21 | F14_R22 | F14_R23 | F14_R24 | F14_R25 | F14_R26 | F14_R27 | F14_R28 | F14_R29 | F14_R30 | F14_R31 | F14_R32 |
| H | F13 | F13_R21 | F13_R22 | F13_R23 | F13_R24 | F13_R25 | F13_R26 | F13_R27 | F13_R28 | F13_R29 | F13_R30 | F13_R31 | F13_R32 |

Note: the paired forward and reverse primers were located on the 96 well plate, each pair is unique.

**The second round PCR primers**

| Name | Sequence |
| --- | --- |
| PCR_Truseq_Amp_F | AATGATACGGCGACCACCGAGATCTACACTCTTTCCCTACACGAC |
| PCR_Truseq_Amp_R_1 | CAAGCAGAAGACGGCATACGAGATCGTGATGTGACTGGAGTTCAGACG |
| PCR_Truseq_Amp_R_2 | CAAGCAGAAGACGGCATACGAGATACATCGGTGACTGGAGTTCAGACG |
| PCR_Truseq_Amp_R_3 | CAAGCAGAAGACGGCATACGAGATGCCTAAGTGACTGGAGTTCAGACG |
| PCR_Truseq_Amp_R_4 | CAAGCAGAAGACGGCATACGAGATTGGTCAGTGACTGGAGTTCAGACG |
| PCR_Truseq_Amp_R_5 | CAAGCAGAAGACGGCATACGAGATCACTGTGTGACTGGAGTTCAGACG |
| PCR_Truseq_Amp_R_6 | CAAGCAGAAGACGGCATACGAGATATTGGCGTGACTGGAGTTCAGACG |
| PCR_Truseq_Amp_R_7 | CAAGCAGAAGACGGCATACGAGATGATCTGGTGACTGGAGTTCAGACG |
| PCR_Truseq_Amp_R_8 | CAAGCAGAAGACGGCATACGAGATTCAAGTGTGACTGGAGTTCAGACG |
| PCR_Truseq_Amp_R_9 | CAAGCAGAAGACGGCATACGAGATCTGATCGTGACTGGAGTTCAGACG |
| PCR_Truseq_Amp_R_10 | CAAGCAGAAGACGGCATACGAGATAAGCTAGTGACTGGAGTTCAGACG |
| PCR_Truseq_Amp_R_11 | CAAGCAGAAGACGGCATACGAGATGTAGCCGTGACTGGAGTTCAGACG |
| PCR_Truseq_Amp_R_12 | CAAGCAGAAGACGGCATACGAGATTACAAGGTGACTGGAGTTCAGACG |
| PCR_Truseq_Amp_R_13 | CAAGCAGAAGACGGCATACGAGATTTGACTGTGACTGGAGTTCAGACG |
| PCR_Truseq_Amp_R_14 | CAAGCAGAAGACGGCATACGAGATGGAACTGTGACTGGAGTTCAGACG |
| PCR_Truseq_Amp_R_15 | CAAGCAGAAGACGGCATACGAGATTGACATGTGACTGGAGTTCAGACG |
| PCR_Truseq_Amp_R_16 | CAAGCAGAAGACGGCATACGAGATGGACGGGTGACTGGAGTTCAGACG |
| PCR_Truseq_Amp_R_18 | CAAGCAGAAGACGGCATACGAGATGCGGACGTGACTGGAGTTCAGACG |
| PCR_Truseq_Amp_R_19 | CAAGCAGAAGACGGCATACGAGATTTTCACGTGACTGGAGTTCAGACG |
| PCR_Truseq_Amp_R_20 | CAAGCAGAAGACGGCATACGAGATGGCCACGTGACTGGAGTTCAGACG |
| PCR_Truseq_Amp_R_21 | CAAGCAGAAGACGGCATACGAGATCGAAACGTGACTGGAGTTCAGACG |
| PCR_Truseq_Amp_R_22 | CAAGCAGAAGACGGCATACGAGATCGTACGGTGACTGGAGTTCAGACG |
| PCR_Truseq_Amp_R_23 | CAAGCAGAAGACGGCATACGAGATCCACTCGTGACTGGAGTTCAGACG |
| PCR_Truseq_Amp_R_25 | CAAGCAGAAGACGGCATACGAGATATCAGTGTGACTGGAGTTCAGACG |
| PCR_Truseq_Amp_R_27 | CAAGCAGAAGACGGCATACGAGATAGGAATGTGACTGGAGTTCAGACG |

Note: The red color upper case letters are Illumina Truseq barcodes, the black color upper case letters are part of Illumina Truseq adapter.

**Table S2. The genotypes of the *TaGW2* gene mutants in the 2017 spring experiment**

| Plant_ID | Genotype | Genome A | Genome B | Genome D |
| --- | --- | --- | --- | --- |
| 239-1-2-1 | aabbdd | 142 -1 | 142 -1 | 142 -1 |
| 239-1-2-2 | aabbdd | 142 -1 | 142 -1 | 142 -1 |
| 239-1-2-3 | aabbdd | 142 -1 | 142 -1 | 142 -1 |
| 239-1-2-4 | aabbdd | 142 -1 | 142 -1 | 142 -1 |
| 239-1-2-5 | aabbdd | 142 -1 | 142 -1 | 142 -1 |
| 299-1-14-1 | aaBBDD | 142 -1 |  |  |
| 299-1-14-2 | aaBBDD | 142 -1 |  |  |
| 299-1-14-5 | aaBBDD | 142 -1 |  |  |
| 299-2-1-15 | aaBBdd | 142 -1 |  | 143 +1T |
| 299-2-1-16 | AABBdd |  |  | 143 +1T |
| 299-2-1-18 | aaBBdd | 142 -1 |  | 143 +1T |
| 299-2-1-3 | aaBBdd | 142 -1 |  | 143 +1T |
| 299-2-1-4 | AABBdd |  |  | 143 +1T |
| 299-2-1-5 | AABBdd |  |  | 143 +1T |
| 299-2-4-13 | AAbbdd |  | 117 -26 | 143 +1T |
| 299-2-4-14 | AAbbDD |  | 117 -26 |  |
| 299-2-4-15 | AAbbdd |  | 117 -26 | 143 +1T |
| 299-2-4-18 | AAbbDD |  | 117 -26 |  |
| 299-2-4-22 | AABBDD |  |  |  |
| 299-2-4-23 | AABBdd |  |  | 143 +1T |
| 299-2-4-32 | AAbbDD |  | 117 -26 |  |
| 299-2-4-33 | AAbbdd |  | 117 -26 |  |
| 299-2-4-4 | AABBDD |  |  |  |
| 299-2-4-40 | AAbbdd |  | 117 -26 | 143 +1T |
| 299-2-4-42 | AAbbDD |  | 117 -26 |  |
| 299-2-4-54 | AABBDD |  |  |  |
| 299-2-4-6 | AABBDD |  |  |  |
| 708-1-13 | aaBBDD | 142 -1 |  |  |
| 708-1-23 | AABBDD |  |  |  |
| 920-1-2 | AABBdd |  |  | 143 +1T |
| 920-1-5 | AABBdd |  |  | 143 +1T |
| 920-1-7 | AABBDD |  |  |  |
| 920-2-2 | AAbbDD |  | 143 +1A |  |
| 920-2-8 | AAbbDD |  | 143 +1A |  |
| Bobwhite-1 | AABBDD |  |  |  |
| Bobwhite-2 | AABBDD |  |  |  |
| Bobwhite-3 | AABBDD |  |  |  |
| Bobwhite-4 | AABBDD |  |  |  |
| Bobwhite-6 | AABBDD |  |  |  |
| Bobwhite-7 | AABBDD |  |  |  |

Note: The A/a, B/b, and D/d in the genotype represent the A, B, and D genome homoeologue respectively. The lower and upper case represent mutant and wild type alleles respectively. The mutations of each mutated allele are shown in the right three columns. The nucleotide “A” in the start codon is assigned as position 1. The start position of each mutation is shown on the left, and it is followed by the deletion or insertion of different number nucleotides. The deletion and insertion is shown as “-” and “+” respectively.

**Table S3. The genotypes of the *TaGW2* gene mutants in the 2017 fall experiment**

| Plant_ID | Genotype | Genome A | Genome B | Genome D |
| --- | --- | --- | --- | --- |
| 1001-1-1-6 | aabbdd | 142 -1 137 -26 | 142 -1 | 142 -1 |
| 1001-1-26 | aabbdd | 134 -7 137 -26 | 142 -1 | 142 -1 |
| 1001-1-52 | aabbdd | 134 -7 137 -26 | 142 -1 143 +1G | 142 -1 |
| 1902-1-14 | aabbdd | 142 -1 | 141 -2+1A | 142 -1 |
| 1902-1-15 | aabbdd | 142 -1 | 141 -2+1A | 142 -1 |
| 1902-1-19 | aabbdd | 142 -1 | 141 -2+1A | 142 -1 |
| 1902-1-22 | AaBbDd | 142 -1 | 141 -2+1A | 142 -1 |
| 1902-1-23 | aabbdd | 142 -1 | 141 -2+1A | 142 -1 |
| 1902-1-31 | aabbdd | 142 -1 | 141 -2+1A | 142 -1 |
| 1902-1-37 | aabbdd | 142 -1 | 141 -2+1A | 142 -1 |
| 239-1-2-1-1 | aabbdd | 142 -1 | 142 -1 | 142 -1 |
| 239-1-2-1-2 | aabbdd | 142 -1 | 142 -1 | 142 -1 |
| 239-1-2-2-1 | aabbdd | 142 -1 | 142 -1 | 142 -1 |
| 239-1-2-2-2 | aabbdd | 142 -1 | 142 -1 | 142 -1 |
| 239-1-2-3-1 | aabbdd | 142 -1 | 142 -1 | 142 -1 |
| 239-1-2-3-2 | aabbdd | 142 -1 | 142 -1 | 142 -1 |
| 239-1-2-4-1 | aabbdd | 142 -1 | 142 -1 | 142 -1 |
| 239-1-2-4-2 | aabbdd | 142 -1 | 142 -1 | 142 -1 |
| 239-1-2-4-3 | aabbdd | 142 -1 | 142 -1 | 142 -1 |
| 239-1-2-5-1 | aabbdd | 142 -1 | 142 -1 | 142 -1 |
| 239-1-2-5-2 | aabbdd | 142 -1 | 142 -1 | 142 -1 |
| 239-1-2-5-3 | aabbdd | 142 -1 | 142 -1 | 142 -1 |
| 299-1-14-1-1 | aaBBDD | 142 -1 |  |  |
| 299-1-14-1-2 | aaBBDD | 142 -1 |  |  |
| 299-1-14-2-2 | aaBBDD | 142 -1 |  |  |
| 299-1-14-3-1 | aaBBDD | 142 -1 |  |  |
| 299-1-14-3-3 | aaBBDD | 142 -1 |  |  |
| 299-2-1-15-1 | aaBBdd | 142 -1 |  | 143 +1T |
| 299-2-1-15-2 | aaBBdd | 142 -1 |  | 143 +1T |
| 299-2-1-15-3 | aaBBdd | 142 -1 |  | 143 +1T |
| 299-2-1-15-6 | aaBBdd | 142 -1 |  | 143 +1T |
| 299-2-1-15-7 | aaBBdd | 142 -1 |  | 143 +1T |
| 299-2-1-18-4 | aaBBdd | 142 -1 |  | 143 +1T |
| 299-2-1-18-5 | aaBBdd | 142 -1 |  | 143 +1T |
| 299-2-1-18-7 | aaBBdd | 142 -1 |  | 143 +1T |
| 299-2-1-18-8 | aaBBdd | 142 -1 |  | 143 +1T |
| 299-2-1-3-1 | aaBBdd | 142 -1 |  | 143 +1T |
| 299-2-1-3-2 | aaBBdd | 142 -1 |  | 143 +1T |
| 299-2-1-3-3 | aaBBdd | 142 -1 |  | 143 +1T |
| 299-2-1-3-4 | aaBBdd | 142 -1 |  | 143 +1T |
| 299-2-1-3-5 | aaBBdd | 142 -1 |  | 143 +1T |
| 299-2-1-3-7 | aaBBdd | 142 -1 |  | 143 +1T |
| 299-2-1-3-8 | aaBBdd | 142 -1 |  | 143 +1T |
| 299-2-1-4-1 | AABBdd |  |  | 143 +1T |
| 299-2-1-4-4 | AABBdd |  |  | 143 +1T |
| 299-2-1-4-5 | AABBdd |  |  | 143 +1T |
| 299-2-1-5-1 | AABBdd |  |  | 143 +1T |
| 299-2-1-5-2 | AABBdd |  |  | 143 +1T |
| 299-2-1-5-4 | AABBdd |  |  | 143 +1T |
| 299-2-4-13-1 | AAbbdd |  | 117 -26 | 143 +1T |
| 299-2-4-13-2 | AAbbdd |  | 117 -26 | 143 +1T |
| 299-2-4-13-3 | AAbbdd |  | 117 -26 | 143 +1T |
| 299-2-4-13-5 | AAbbdd |  | 117 -26 | 143 +1T |
| 299-2-4-13-6 | AAbbdd |  | 117 -26 | 143 +1T |
| 299-2-4-13-7 | AAbbdd |  | 117 -26 | 143 +1T |
| 299-2-4-13-8 | AAbbdd |  | 117 -26 | 143 +1T |
| 299-2-4-14-2 | AAbbDD |  | 117 -26 |  |
| 299-2-4-14-3 | AAbbDD |  | 117 -26 |  |
| 299-2-4-15-2 | AAbbdd |  | 117 -26 | 143 +1T |
| 299-2-4-15-3 | AAbbdd |  | 117 -26 | 143 +1T |
| 299-2-4-15-4 | AAbbdd |  | 117 -26 | 143 +1T |
| 299-2-4-15-5 | AAbbdd |  | 117 -26 | 143 +1T |
| 299-2-4-15-7 | AAbbdd |  | 117 -26 | 143 +1T |
| 299-2-4-15-8 | AAbbdd |  | 117 -26 | 143 +1T |
| 299-2-4-18-2 | AAbbDD |  | 117 -26 |  |
| 299-2-4-18-3 | AAbbDD |  | 117 -26 |  |
| 299-2-4-18-4 | AAbbDD |  | 117 -26 |  |
| 299-2-4-22-1 | AABBDD |  |  |  |
| 299-2-4-22-2 | AABBDD |  |  |  |
| 299-2-4-22-3 | AABBDD |  |  |  |
| 299-2-4-23-1 | AABBdd |  |  | 143 +1T |
| 299-2-4-23-2 | AABBdd |  |  | 143 +1T |
| 299-2-4-23-3 | AABBdd |  |  | 143 +1T |
| 299-2-4-23-4 | AABbdd |  |  | 143 +1T |
| 299-2-4-23-5 | AABBdd |  |  | 143 +1T |
| 299-2-4-32-1 | AAbbDD |  | 117 -26 |  |
| 299-2-4-32-2 | AAbbDD |  | 117 -26 |  |
| 299-2-4-32-3 | AAbbDD |  | 117 -26 |  |
| 299-2-4-32-4 | AAbbDD |  | 117 -26 |  |
| 299-2-4-33-1 | AAbbdd |  | 117 -26 | 143 +1T |
| 299-2-4-33-2 | AAbbdd |  | 117 -26 | 143 +1T |
| 299-2-4-33-3 | AAbbdd |  | 117 -26 | 143 +1T |
| 299-2-4-33-4 | AAbbdd |  | 117 -26 | 143 +1T |
| 299-2-4-40-1 | AAbbdd |  | 117 -26 | 143 +1T |
| 299-2-4-40-2 | AAbbdd |  | 117 -26 | 143 +1T |
| 299-2-4-40-3 | AAbbdd |  | 117 -26 | 143 +1T |
| 299-2-4-40-5 | AAbbdd |  | 117 -26 | 143 +1T |
| 299-2-4-40-6 | AAbbdd |  | 117 -26 | 143 +1T |
| 299-2-4-40-7 | AAbbdd |  | 117 -26 | 143 +1T |
| 299-2-4-4-1 | AABBDD |  |  |  |
| 299-2-4-4-2 | AABBDD |  |  |  |
| 299-2-4-42-1 | AAbbDD |  | 117 -26 |  |
| 299-2-4-42-2 | AAbbDD |  | 117 -26 |  |
| 299-2-4-42-3 | AAbbDD |  | 117 -26 |  |
| 299-2-4-42-4 | AAbbDD |  | 117 -26 |  |
| 299-2-4-4-3 | AABBDD |  |  |  |
| 299-2-4-4-4 | AABBDD |  |  |  |
| 299-2-4-4-5 | AABBDD |  |  |  |
| 299-2-4-4-6 | AABBDD |  |  |  |
| 299-2-4-49-3 | AAbbdd |  | 117 -26 | 143 +1T |
| 299-2-4-49-5 | AabbDD | 129 -14 | 117 -26 |  |
| 299-2-4-49-6 | AabbDd | 129 -14 | 117 -26 | 143 +1T |
| 299-2-4-49-7 | AAbbDd |  | 117 -26 | 143 +1T |
| 299-2-4-49-8 | AabbDD | 129 -14 | 117 -26 |  |
| 299-2-4-49-11 | AabbDd | 129 -14 | 117 -26 | 143 +1T |
| 299-2-4-49-14 | AabbDd | 129 -14 | 117 -26 | 143 +1T |
| 299-2-4-49-15 | AAbbDd |  | 117 -26 | 143 +1T |
| 299-2-4-49-16 | aabbDd | 129 -14 | 117 -26 | 143 +1T |
| 299-2-4-49-17 | AAbbDd |  | 117 -26 | 143 +1T |
| 299-2-4-49-20 | AAbbDd |  | 117 -26 | 143 +1T |
| 299-2-4-49-23 | aabbDD | 129 -14 | 117 -26 |  |
| 299-2-4-49-24 | AAbbdd |  | 117 -26 | 143 +1T |
| 299-2-4-49-25 | AAbbdd |  | 117 -26 | 143 +1T |
| 299-2-4-49-26 | AAbbDd |  | 117 -26 | 143 +1T |
| 299-2-4-49-28 | Aabbdd | 129 -14 | 117 -26 | 143 +1T |
| 299-2-4-49-33 | AAbbDd |  | 117 -26 | 143 +1T |
| 299-2-4-49-36 | AAbbDd |  | 117 -26 | 143 +1T |
| 299-2-4-49-38 | AAbbDd |  | 117 -26 | 143 +1T |
| 299-2-4-49-39 | AabbDd | 129 -14 | 117 -26 | 143 +1T |
| 299-2-4-49-41 | AAbbdd |  | 117 -26 | 143 +1T |
| 299-2-4-49-44 | AAbbdd |  | 117 -26 | 143 +1T |
| 299-2-4-49-49 | AabbDd | 129 -14 | 117 -26 |  |
| 299-2-4-49-50 | AAbbDD |  | 117 -26 |  |
| 299-2-4-49-51 | AabbDd | 129 -14 | 117 -26 | 143 +1T |
| 299-2-4-49-52 | AAbbDD |  | 117 -26 |  |
| 299-2-4-49-53 | Aabbdd |  | 117 -26 | 143 +1T |
| 299-2-4-49-54 | AAbbdd |  | 117 -26 | 143 +1T |
| 299-2-4-49-55 | AabbDd |  | 117 -26 | 143 +1T |
| 299-2-4-49-57 | aabbDD | 129 -14 | 117 -26 |  |
| 299-2-4-49-58 | AAbbdd |  | 117 -26 | 143 +1T |
| 299-2-4-49-59 | AAbbDD |  | 117 -26 |  |
| 299-2-4-54-1 | AABBDD |  |  |  |
| 299-2-4-54-2 | AABBDD |  |  |  |
| 299-2-4-6-1 | AABBDD |  |  |  |
| 299-2-4-6-2 | AABBDD |  |  |  |
| 299-2-4-6-3 | AABBDD |  |  |  |
| 314-1-3-13-1 | AABBDD |  |  |  |
| 314-1-3-13-2 | AaBBDD | 117 -26 |  |  |
| 314-1-3-13-3 | AABBDD |  |  |  |
| 314-1-3-13-4 | AABBDD |  |  |  |
| 314-1-3-15-1 | aaBbDd | 142 -1 | 129 -13 | 142 -1 |
| 314-1-3-15-2 | aaBBDD | 142 -1 |  |  |
| 314-1-3-15-3 | aaBBDD | 142 -1 |  |  |
| 314-1-3-16-1 | aaBBDD | 142 -1 |  |  |
| 314-1-3-16-2 | aaBBDD | 142 -1 |  |  |
| 314-1-3-16-3 | aaBBDD | 142 -1 |  |  |
| 314-1-3-17-1 | AABBDD |  |  |  |
| 314-1-3-17-3 | AABBDD |  |  |  |
| 314-1-3-19-1 | aaBBDD | 142 -1 |  |  |
| 314-1-3-19-2 | aaBBDD | 142 -1 |  |  |
| 314-1-3-19-3 | aaBBDD | 142 -1 |  |  |
| 314-1-3-3-1 | aaBBDD | 142 -1 |  |  |
| 314-1-3-3-2 | aaBBDD | 142 -1 |  |  |
| 314-1-3-3-3 | aaBBDD | 142 -1 |  |  |
| 314-1-3-6-1 | AABBDD |  |  |  |
| 314-1-3-6-2 | AABBDD |  |  |  |
| 314-1-3-6-3 | AABBDD |  |  |  |
| 314-1-3-6-3 | AABBDD |  |  |  |
| 314-1-3-6-4 | AABBDD |  |  |  |
| 314-1-3-8-4 | AabbDD | 142 -1 | 117 -26 143 +1A |  |
| 314-1-3-8-5 | AaBbDd | 142 -1 | 136 -11 142 -1 117 -26 | 142 -1 |
| 314-1-3-8-7 | aaBbDD | 142 -1 | 143 +1A |  |
| 314-1-3-8-9 | AabbDD | 142 -1 | 143 +1A 145 -11+6TGTACA | |
| 314-1-3-8-10 | AABbDD |  | 143 +1A |  |
| 314-1-3-8-12 | aaBbDD | 142 -1 | 141 -2 |  |
| 314-1-3-8-13 | AaBbDD | 142 -1 | 143 +1A |  |
| 314-1-3-8-14 | AaBbDd | 142 -1 | 117 -26 | 143 +1T |
| 314-1-3-8-16 | aaBBDD | 142 -1 |  |  |
| 314-1-3-8-18 | AabbDD | 142 -1 | 143 +1A |  |
| 314-1-3-8-19 | aaBbDD | 142 -1 | 117 -26 |  |
| 314-1-3-8-22 | AABBDD |  |  |  |
| 314-1-3-8-23 | AabbDD | 142 -1 | 143 +1A |  |
| 314-1-3-8-24 | aabbDD | 142 -1 | 143 +1A |  |
| 314-1-3-8-25 | AaBbDD | 142 -1 | 143 +1A |  |
| 314-1-3-8-26 | AaBBDD | 142 -1 |  |  |
| 314-1-3-8-27 | AaBBDD | 142 -1 |  |  |
| 314-1-3-8-28 | AaBbDD | 142 -1 | 143 +1A |  |
| 314-1-3-8-29 | AabbDD | 142 -1 | 143 +1A |  |
| 314-1-3-8-30 | AabbDD | 142 -1 | 143 +1A |  |
| 314-1-3-8-31 | AABbDD |  | 143 +1A |  |
| 314-1-3-8-32 | aaBbDD | 142 -1 | 143 +1A |  |
| 314-1-3-8-33 | aabbDD | 142 -1 | 143 +1A |  |
| 314-1-3-8-34 | aaBbDD | 142 -1 | 117 -26 |  |
| 314-1-3-8-35 | AabbDD | 142 -1 | 143 +1A |  |
| 314-1-3-8-36 | aabbDD | 142 -1 | 143 +1A |  |
| 314-1-3-8-37 | AabbDD | 142 -1 | 117 -26 143 +1A |  |
| 314-1-3-8-38 | AAbbDD |  | 143 +1A |  |
| 314-1-3-8-39 | AaBbDD | 142 -1 | 143 +1A |  |
| 314-1-3-8-40 | AaBBDD | 142 -1 |  |  |
| 314-1-3-8-41 | AaBbDD | 142 -1 | 143 +1A |  |
| 314-1-3-8-42 | AABbDD |  | 117 -26 |  |
| 314-1-3-8-43 | AaBbDD | 142 -1 | 143 +1A |  |
| 314-1-3-8-45 | AaBbDD | 142 -1 | 143 +1A |  |
| 314-1-3-8-46 | AaBbDD | 142 -1 | 143 +1A |  |
| 314-1-3-8-51 | AaBbDD | 142 -1 | 143 +1A |  |
| 314-1-3-8-53 | AabbDD | 142 -1 | 143 +1A |  |
| 314-1-3-8-54 | AabbDD | 142 -1 | 143 +1A |  |
| 314-1-3-8-55 | AabbDD | 142 -1 | 143 +1A |  |
| 314-1-3-8-56 | aaBbDD | 142 -1 | 143 +1A |  |
| 314-1-3-8-57 | AABbDD |  |  |  |
| 314-1-3-8-58 | AaBbDD | 142 -1 | 143 +1A |  |
| 314-1-3-8-59 | AabbDD | 142 -1 | 143 +1A |  |
| 314-1-3-8-60 | AabbDD | 142 -1 | 143 +1A |  |
| 314-1-3-8-61 | AaBbDD | 142 -1 133 -17+22AGGGGCTGTACACGAGGCCGCA | 143 +1A |  |
| 314-1-3-8-62 | AaBbDD | 142 -1 | 143 +1A |  |
| 314-1-3-8-64 | AAbbDD |  | 143 +1A |  |
| 314-1-3-8-65 | AABbDD |  | 143 +1A |  |
| 314-1-3-8-69 | AABbDD |  | 143 +1A |  |
| 314-1-3-8-70 | AabbDd | 142 -1 | 143 +1A | 143 +1A/C |
| 314-1-3-8-71 | AabbDD | 142 -1 | 143 +1A |  |
| 314-1-3-8-72 | aabbDD | 142 -1 | 143 +1A |  |
| 314-1-3-8-73 | AaBbDD | 142 -1 | 143 +1A |  |
| 314-1-3-8-74 | aabbDD | 142 -1 | 143 +1A |  |
| 314-1-3-8-77 | AabbDD | 142 -1 | 143 +1A |  |
| 314-1-3-8-78 | AaBbDD | 142 -1 | 143 +1A |  |
| 314-1-3-8-79 | AaBBDD | 142 -1 |  |  |
| 314-1-3-8-81 | AaBbDD | 142 -1 |  |  |
| 314-1-3-8-82 | AAbbDD |  | 143 +1A |  |
| 314-1-3-8-83 | aaBBDD | 142 -1 |  |  |
| 314-1-3-8-84 | AaBbDD | 142 -1 | 143 +1A |  |
| 314-1-3-9-1 | AABbDD |  | 142 -1 |  |
| 314-1-3-9-2 | AABBDD |  |  |  |
| 707-1-4-2 | AAbbDD |  | 143 +1A |  |
| 707-1-4-4 | AAbbDD |  | 136 -11 |  |
| 707-1-4-5 | AAbbDD |  | 136 -11 |  |
| 707-1-7-1 | AAbbDD |  | 136 -11 |  |
| 707-1-7-2 | AAbbDD |  | 136 -11 |  |
| 707-1-7-3 | AAbbDD |  | 136 -11 |  |
| 707-1-7-4 | AAbbDD |  | 136 -11 |  |
| 707-1-7-5 | AAbbDD |  | 136 -11 |  |
| 707-1-11-2 | AAbbDD |  | 136 -11 |  |
| 707-1-11-3 | AAbbDD |  | 136 -11 |  |
| 707-1-11-4 | AAbbDD |  | 136 -11 |  |
| 707-1-11-6 | AAbbDD |  | 136 -11 |  |
| 707-1-17-2 | aabbdd | 142 -1 143 +1T | 130 -16 143 +1T | 143 +1T/A 141 -2 |
| 707-1-17-3 | aabbdd | 142 -1 | 142 -1 | 143 +1G 141 -2 |
| 707-1-17-4 | aabbdd | 143 +1T | 142 -1 143 +1A | 142 -1 141 -2 |
| 707-1-22-1 | aabbdd | 142 -1 143 +1A | 143 +1C | 142 -1 |
| 707-1-22-3 | aabbdd | 142 -1 | 136 -11 117 -26 142 -1 | 142 -1 |
| 707-1-26 | AabbDd | 142 -1 143 +1A/G/T | 136 -11 | 142 -1 143 +1A/T |
| 707-1-27 | AaBbDd | 142 -1 143 +1A/T/C/G | 136 -11 | 143 +1A 140 -5+40TCCTGGTAACAGATCTTTATCCTCTCCCCAGTCTCTCCCC |
| 707-1-28 | AabbDd | 142 -1 | 136 -11 | 142 -1 |
| 707-1-30 | AabbDd | 142 -1 | 136 -11 | 142 -1 |
| 707-1-31 | AabbDd | 142 -1 142 -7 141 -2 | 136 -11 | 142 -1 143 +1A/T |
| 707-1-32 | AAbbDD |  | 136 -11 |  |
| 707-1-34 | AAbbDd |  | 136 -11 | 143 +1C |
| 707-1-36 | AAbbDD |  | 136 -11 |  |
| 707-1-38 | aabbDd | 142 -1 143 +15TGCTACCTGCTACCT | 136 -11 117 -26 | 141 -2 142 -1 143 +1G/A/T |
| 707-1-39 | AaBbDd | 142 -1 | 136 -11 | 143 +1T |
| 707-1-40 | AabbDD | 142 -1 | 136 -11 |  |
| 707-1-41 | AaBbDD | 142 -1 136 -11 | 136 -11 142 -1 |  |
| 707-1-42 | AABbDD |  | 136 -11 |  |
| 707-1-44 | AABbDD |  | 136 -11 |  |
| 707-1-46 | AaBbDd | 142 -1 | 143 +1T/A/G 142 -1 | 141 -2 |
| 707-1-49 | aabbDd | 140 -5 142 -1 143 +1A/T 136 -11 | 136 -11 142 -1 | 142 -1 143 +14TGGTAACAGGTCTTTATCCAATGG 143 +1T/A |
| 707-1-51 | AAbbDD |  | 136 -11 |  |
| 707-1-52 | AAbbDD |  | 136 -11 |  |
| 707-1-53 | aabbdd | 142 -1 137 -7 115 -26 | 136 -11 142 -1 143 +1A/T | 142 -1 143 +1A/T |
| 707-1-54 | aabbdd | 142 -1 143 +1C/A 136 -11 | 136 -11 142 -1 | 143 +1A 142 -1 136 -11 137 -11 |
| 707-1-55 | aabbdd | 143 +1A/T | 136 -11 142 -1 | 138 -4 |
| 707-1-56 | aabbdd | 142 -1 143 +1A/T/G 138 -4 | 136 -11 142 -1 143 +1A/T | 142 -1 133 -8 |
| 707-1-58 | aabbdd | 142 -1 | 143 +1A/T 142 -1 | 142 -1 143 +1A/G/T 141 -2 |
| 707-1-60 | aabbdd | 142 -1 | 136 -11 | 143 +1A |
| 707-1-61 | AABbDD |  | 136 -11 |  |
| 707-1-62 | AAbbDD |  | 136 -11 |  |
| 707-1-63 | aabbdd | 142 -1 143 +1T | 136 -11 142 -1 | 142 -1 143 +1A/T/C/G |
| 707-1-65 | AabbDd |  | 136 -11 |  |
| 707-1-66 | aabbdd | 140 -20 143 +1T | 142 -1 117 -26 | 142 -1 143 +1T/A 117 -26 |
| 707-1-67 | aaBbDd | 142 -1 143 +1A/T/G | 136 -11 | 142 -1 |
| 707-1-68 | AAbbDD |  | 136 -11 142 -1 |  |
| 707-1-69 | AAbbDD |  | 136 -11 |  |
| 707-1-70 | aabbdd | 142 -1 141 -11 | 136 -11 | 141 -4 142 -1 143 +1T/A |
| 707-1-71 | aaBbdd | 142 -1 | 142 -1 141 -2 | 142 -1 140 -10 |
| 707-1-72 | AabbDd | 143 +1C | 136 -11 | 142 -1 143 +1A/T/G |
| 707-1-73 | AabbDD | 142 -1 143 +1T | 136 -11 142 -1 |  |
| 707-1-74 | AaBbDD | 143 +1T | 136 -11 |  |
| 707-1-76 | AaBbDD | 142 -1 143 +1A/T | 136 -11 142 -1 |  |
| 707-1-77 | aabbdd | 142 -1 143 +1A/T | 136 -11 | 142 -1 |
| 707-1-78 | aaBbDd | 143 +1T | 136 -11 | 142 -1 |
| 707-1-79 | aabbdd | 142 -1 143 +1C | 141 -5 143 +1A/C/T | 143 +1A 142 -1 |
| 707-1-80 | AabbDd | 138 -5 142 -1 141 -2 | 136 -11 143 +1T | 142 -1 143 +1T |
| 707-1-81 | AaBBDD | 142 -1 141-2 143 +1A/T/G | |  |
| 707-1-82 | AaBbDd | 142 -1 | 142 -1 | 142 -1 141-2 143 +1T/A |
| 707-1-87 | AabbDd | 142 -1 | 136 -11 142 -1 | 142 -1 |
| 707-1-90 | aabbDd | 143 +1A/T/C/G 142 -1 141 -2 | 136 -11 | 142 -1 |
| 707-1-91 | aabbDd | 141 -2 142-1 | 136 -11 142 -1 | 138 -4 142 -1 141 -2 143 +1T/G |
| 708-1-13-1 | aaBBDD | 142 -1 |  |  |
| 708-1-13-2 | aaBBDD | 142 -1 |  |  |
| 708-1-13-3 | aaBBDD | 142 -1 |  |  |
| 708-1-13-4 | aaBBDD | 142 -1 |  |  |
| 708-1-14-1 | AAbbDD |  | 117 -26 |  |
| 708-1-14-2 | AAbbDD |  | 117 -26 |  |
| 708-1-14-3 | AAbbDD |  | 117 -26 |  |
| 708-1-14-4 | AAbbDD |  | 117 -26 |  |
| 708-1-23-10 | AABBDD |  |  |  |
| 708-1-23-11 | AABBDD |  |  |  |
| 708-1-23-4 | AABBDD |  |  |  |
| 708-1-23-5 | AABBDD |  |  |  |
| 708-1-23-7 | AABBDD |  |  |  |
| 708-1-23-9 | AABBDD |  |  |  |
| 708-1-4-1 | aaBBDD | 142 -1 |  |  |
| 708-1-4-4 | aaBBDD | 142 -1 |  |  |
| 920-1-2-1 | AABBdd |  |  | 143 +1T |
| 920-1-2-10 | AABBdd |  |  | 143 +1T |
| 920-1-2-11 | AABBdd |  |  | 143 +1T |
| 920-1-2-2 | AABBdd |  |  | 143 +1T |
| 920-1-2-3 | AABBdd |  |  | 143 +1T |
| 920-1-2-4 | AABBDd |  |  | 143 +1T |
| 920-1-2-5 | AABBdd |  |  | 143 +1T |
| 920-1-2-6 | AABBdd |  |  | 143 +1T |
| 920-1-2-7 | AABBdd |  |  | 143 +1T |
| 920-1-2-8 | AABBdd |  |  | 143 +1T |
| 920-1-2-9 | AABBdd |  |  | 143 +1T |
| 920-1-5-1 | AABBdd |  |  | 143 +1T |
| 920-1-5-10 | AABBdd |  |  | 143 +1T |
| 920-1-5-11 | AABBdd |  |  | 143 +1T |
| 920-1-5-3 | AABBdd |  |  | 143 +1T |
| 920-1-5-4 | AABBdd |  |  | 143 +1T |
| 920-1-5-6 | AABBdd |  |  | 143 +1T |
| 920-1-5-9 | AABBdd |  |  | 143 +1T |
| 920-1-7-1 | AABBDD |  |  |  |
| 920-1-7-2 | AABBDD |  |  |  |
| 920-1-7-3 | AABBDD |  |  |  |
| 920-1-7-4 | AABBDD |  |  |  |
| 920-2-14-1 | AABbDD |  | 143 +1A |  |
| 920-2-14-2 | AABbDD |  | 143 +1A |  |
| 920-2-14-3 | AABbDD |  | 143 +1A |  |
| 920-2-2-1 | AAbbDD |  | 143 +1A |  |
| 920-2-2-2 | AAbbDD |  | 143 +1A |  |
| 920-2-2-3 | AAbbDD |  | 143 +1A |  |
| 920-2-2-4 | AAbbDD |  | 143 +1A |  |
| 920-2-2-5 | AABbDD |  | 143 +1A |  |
| 920-2-2-7 | AAbbDD |  | 143 +1A |  |
| 920-2-2-8 | AAbbDD |  | 143 +1A |  |
| 920-2-8-2 | AAbbDD |  | 143 +1A |  |
| 920-2-8-3 | AAbbDD |  | 143 +1A |  |
| 920-2-8-4 | AAbbDD |  | 143 +1A |  |
| 920-2-8-8 | AAbbDD |  | 143 +1A |  |
| 920-2-9-2 | AABbDD |  | 143 +1A |  |
| 920-2-9-3 | AAbbDD |  | 143 +1A |  |
| 920-2-9-4 | AABBDD |  |  |  |
| BW/239-1-12F1-1-34 | aaBBDD | 142 -1 |  |  |
| BW/239-1-12F1-1-35 | aaBBDd | 142 -1 |  | 142 -1 |
| BW/239-1-12F1-1-37 | AaBBDD | 142 -1 |  |  |
| BW/239-1-12F1-2-13 | AaBBDd | 142 -1 |  | 142 -1 |
| BW/239-1-12F1-2-14 | AABBDd | 142 -1 |  | 142 -1 |
| BW/239-1-12F1-2-28 | AaBBdd | 142 -1 |  | 142 -1 |
| BW/239-1-12F1-2-3 | AABBdd |  |  | 142 -1 |
| BW/239-1-12F1-2-5 | aaBBDD | 142 -1 |  |  |
| BW/239-1-12F1-2-6 | aaBBDd | 142 -1 |  | 142 -1 |
| BW-1 | AABBDD |  |  |  |
| BW-10 | AABBDD |  |  |  |
| BW-11 | AABBDD |  |  |  |
| BW-13 | AABBDD |  |  |  |
| BW-15 | AABBDD |  |  |  |
| BW-2 | AABBDD |  |  |  |
| BW-3 | AABBDD |  |  |  |
| BW-4 | AABBDD |  |  |  |
| BW-5 | AABBDD |  |  |  |
| BW-7 | AABBDD |  |  |  |
| BW-8 | AABBDD |  |  |  |

Note: The A/a, B/b, and D/d in the genotype represent the A, B, and D genome homoeologue respectively. The lower and upper case represent mutant and wild type alleles respectively. The mutations of each mutated allele are shown in the right three columns. The nucleotide “A” in the start codon of CDS is assigned as position 1. The start position of each mutation is shown on the left, and it is followed by the deletion or insertion of different number nucleotides. The deletion and insertion is shown as “-” and “+” respectively. If the mutations on one homoeologue are biallelic, both are shown. If one homoeologue has three or more mutation types, the mutations are shown from higher to lower frequency.

**Table S4. The genotypes of the *TaGW2* gene in the Bobwhite and Thatcher F_2_ population**

| Plant_ID | Genotype | Genome A | Genome B | Genome D |
| --- | --- | --- | --- | --- |
| Thatcher/239-1F1-10-3 | aaBBdd | 142 -1 |  | 142 -1 |
| Thatcher/239-1F1-10-4 | aaBBDd | 142 -1 |  | 142 -1 |
| Thatcher/239-1F1-1-14 | AaBBDD | 142 -1 |  |  |
| Thatcher/239-1F1-1-15 | AaBBdd | 142 -1 |  | 142 -1 |
| Thatcher/239-1F1-1-30 | aaBBDd | 142 -1 |  | 142 -1 |
| Thatcher/239-1F1-1-41 | AaBBdd | 142 -1 |  | 142 -1 |
| Thatcher/239-1F1-1-48 | AABBDd |  |  | 142 -1 |
| Thatcher/239-1F1-1-50 | AaBBDd | 142 -1 |  | 142 -1 |
| Thatcher/239-1F1-1-53 | AABBDD |  |  |  |
| Thatcher/239-1F1-1-60 | AaBBdd | 142 -1 |  | 142 -1 |
| Thatcher/239-1F1-1-65 | AABBDd |  |  | 143 +1T |
| Thatcher/239-1F1-1-78 | AaBBDd | 142 -1 |  | 142 -1 |
| Thatcher/239-1F1-1-79 | aaBBdd | 142 -1 |  | 142 -1 |
| Thatcher/239-1F1-1-81 | AaBBDd | 142 -1 |  | 142 -1 |
| Thatcher/239-1F1-1-83 | aaBBDD | 142 -1 |  |  |
| Thatcher/239-1F1-1-86 | AaBBDd | 142 -1 |  | 142 -1 |
| Thatcher/239-1F1-1-89 | AABBDd |  |  | 143 +1T |
| Thatcher/239-1F1-1-93 | AaBBdd | 142 -1 |  | 142 -1 |
| Thatcher/239-1F1-2-1 | AABBDd |  |  | 142 -1 |
| Thatcher/239-1F1-2-10 | AABBdd |  |  | 142 -1 |
| Thatcher/239-1F1-2-2 | aaBBDd | 142 -1 |  | 142 -1 |
| Thatcher/239-1F1-2-23 | aaBBDD | 142 -1 |  |  |
| Thatcher/239-1F1-2-24 | AaBBDd | 142 -1 |  | 142 -1 |
| Thatcher/239-1F1-2-25 | aaBBDd | 142 -1 |  | 142 -1 |
| Thatcher/239-1F1-2-26 | aaBBDd | 142 -1 |  | 142 -1 |
| Thatcher/239-1F1-2-33 | AABBDd |  |  | 142 -1 |
| Thatcher/239-1F1-2-36 | aaBBDd | 142 -1 |  | 142 -1 |
| Thatcher/239-1F1-2-37 | AABBDD |  |  |  |
| Thatcher/239-1F1-2-4 | AaBBdd | 142 -1 |  | 142 -1 |
| Thatcher/239-1F1-2-41 | AaBBdd | 142 -1 |  | 142 -1 |
| Thatcher/239-1F1-2-43 | AaBBDD | 142 -1 |  |  |
| Thatcher/239-1F1-2-60 | AABBDd |  |  | 142 -1 |
| Thatcher/239-1F1-2-77 | AABBDd |  |  | 142 -1 |
| Thatcher/239-1F1-3-1 | aaBbDD | 142 -1 | 142 -1 |  |
| Thatcher/239-1F1-3-10 | AAbbDd |  | 142 -1 | 142 -1 |
| Thatcher/239-1F1-3-11 | AABbDD |  | 142 -1 |  |
| Thatcher/239-1F1-3-13 | AaBbDd | 142 -1 | 142 -1 | 142 -1 |
| Thatcher/239-1F1-3-14 | AABBDd |  |  | 142 -1 |
| Thatcher/239-1F1-3-16 | aaBbDD | 142 -1 | 142 -1 |  |
| Thatcher/239-1F1-3-17 | aabbDd | 142 -1 | 142 -1 | 142 -1 |
| Thatcher/239-1F1-3-18 | AaBbdd | 142 -1 | 142 -1 | 142 -1 |
| Thatcher/239-1F1-3-19 | AABbDd |  | 142 -1 | 142 -1 |
| Thatcher/239-1F1-3-2 | AAbbDd |  | 142 -1 | 142 -1 |
| Thatcher/239-1F1-3-20 | AaBbDd | 142 -1 | 142 -1 | 142 -1 |
| Thatcher/239-1F1-3-21 | AABbDd |  | 142 -1 | 142 -1 |
| Thatcher/239-1F1-3-22 | AaBbDd | 142 -1 | 142 -1 | 142 -1 |
| Thatcher/239-1F1-3-23 | aaBbDd | 142 -1 | 142 -1 | 142 -1 |
| Thatcher/239-1F1-3-24 | aaBbDd | 142 -1 | 142 -1 | 142 -1 |
| Thatcher/239-1F1-3-25 | AABBDd |  |  | 142 -1 |
| Thatcher/239-1F1-3-26 | aabbDD | 142 -1 | 142 -1 |  |
| Thatcher/239-1F1-3-27 | AaBbDD | 142 -1 | 142 -1 |  |
| Thatcher/239-1F1-3-28 | AaBbDD | 142 -1 | 142 -1 |  |
| Thatcher/239-1F1-3-30 | AabbDd | 142 -1 | 142 -1 | 142 -1 |
| Thatcher/239-1F1-3-31 | AAbbdd |  | 142 -1 | 142 -1 |
| Thatcher/239-1F1-3-32 | AabbDd | 142 -1 | 142 -1 | 142 -1 |
| Thatcher/239-1F1-3-33 | AabbDd | 142 -1 | 142 -1 | 142 -1 |
| Thatcher/239-1F1-3-35 | AabbDD | 142 -1 | 142 -1 |  |
| Thatcher/239-1F1-3-38 | AaBBDd | 142 -1 |  | 142 -1 |
| Thatcher/239-1F1-3-39 | AABbDD |  | 142 -1 |  |
| Thatcher/239-1F1-3-4 | AaBBDD | 142 -1 |  |  |
| Thatcher/239-1F1-3-41 | aaBbDd | 142 -1 | 142 -1 | 142 -1 |
| Thatcher/239-1F1-3-42 | aaBbDd | 142 -1 | 142 -1 | 142 -1 |
| Thatcher/239-1F1-3-43 | AaBbDd | 142 -1 | 142 -1 | 142 -1 |
| Thatcher/239-1F1-3-45 | AABbDD |  | 142 -1 |  |
| Thatcher/239-1F1-3-46 | AABbDd |  | 142 -1 | 142 -1 |
| Thatcher/239-1F1-3-47 | AaBbDD | 142 -1 | 142 -1 |  |
| Thatcher/239-1F1-3-48 | AaBbDd | 142 -1 | 142 -1 | 142 -1 |
| Thatcher/239-1F1-3-49 | AaBbDd | 142 -1 | 142 -1 | 142 -1 |
| Thatcher/239-1F1-3-5 | AaBbDd | 142 -1 | 142 -1 | 142 -1 |
| Thatcher/239-1F1-3-50 | AABbDD |  | 142 -1 |  |
| Thatcher/239-1F1-3-51 | aabbDd | 142 -1 | 142 -1 | 142 -1 |
| Thatcher/239-1F1-3-52 | aaBbDd | 142 -1 | 142 -1 | 142 -1 |
| Thatcher/239-1F1-3-53 | aabbDd | 142 -1 | 142 -1 | 142 -1 |
| Thatcher/239-1F1-3-54 | aaBbDd | 142 -1 | 142 -1 | 142 -1 |
| Thatcher/239-1F1-3-55 | aaBBDD | 142 -1 |  |  |
| Thatcher/239-1F1-3-56 | aaBbdd | 142 -1 | 142 -1 | 142 -1 |
| Thatcher/239-1F1-3-57 | AaBbDD | 142 -1 | 142 -1 |  |
| Thatcher/239-1F1-3-58 | aabbDD | 142 -1 | 142 -1 |  |
| Thatcher/239-1F1-3-59 | AaBbDd | 142 -1 | 142 -1 | 142 -1 |
| Thatcher/239-1F1-3-6 | AaBBDD | 142 -1 |  |  |
| Thatcher/239-1F1-3-60 | AaBBDd | 142 -1 |  | 142 -1 |
| Thatcher/239-1F1-3-61 | aaBBDD | 142 -1 |  |  |
| Thatcher/239-1F1-3-62 | AaBbDD | 142 -1 | 142 -1 |  |
| Thatcher/239-1F1-3-63 | aaBbDd | 142 -1 | 142 -1 | 142 -1 |
| Thatcher/239-1F1-3-65 | AabbDd | 142 -1 | 142 -1 | 142 -1 |
| Thatcher/239-1F1-3-66 | aaBbDd | 142 -1 | 142 -1 | 142 -1 |
| Thatcher/239-1F1-3-67 | AABbDD |  | 142 -1 |  |
| Thatcher/239-1F1-3-68 | AaBbDd | 142 -1 | 142 -1 | 142 -1 |
| Thatcher/239-1F1-3-69 | AABbDD |  | 142 -1 |  |
| Thatcher/239-1F1-3-7 | AaBbDd | 142 -1 | 142 -1 | 142 -1 |
| Thatcher/239-1F1-3-70 | AaBbDd | 142 -1 | 142 -1 | 142 -1 |
| Thatcher/239-1F1-3-71 | AabbDd | 142 -1 | 142 -1 | 142 -1 |
| Thatcher/239-1F1-3-8 | aaBbDD | 142 -1 | 142 -1 |  |
| Thatcher/239-1F1-3-9 | aabbdd | 142 -1 | 142 -1 | 142 -1 |
| Thatcher/239-1F1-4-16 | AABBDd |  |  | 142 -1 |
| Thatcher/239-1F1-4-34 | AaBBDd | 142 -1 |  | 142 -1 |
| Thatcher/239-1F1-51-18 | AaBBdd | 142 -1 |  | 142 -1 |
| Thatcher/239-1F1-51-44 | AABBDd |  |  | 142 -1 |
| Thatcher/239-1F1-51-5 | AaBBDd | 142 -1 |  | 142 -1 |
| Thatcher/239-1F1-51-53 | aaBBDd | 142 -1 |  | 142 -1 |
| Thatcher/239-1F1-51-59 | aaBBDd | 142 -1 |  | 142 -1 |
| Thatcher/239-1F1-51-62 | AABBdd |  |  | 142 -1 |
| Thatcher/239-1F1-51-8 | AaBBdd | 142 -1 |  | 142 -1 |
| Thatcher/239-1F1-51-9 | AaBBDd | 142 -1 |  | 142 -1 |
| Thatcher/239-1F1-52-1 | AABbDd |  | 142 -1 | 142 -1 |
| Thatcher/239-1F1-52-10 | AaBbdd | 142 -1 | 142 -1 | 142 -1 |
| Thatcher/239-1F1-52-11 | aaBbDd | 142 -1 | 142 -1 | 142 -1 |
| Thatcher/239-1F1-52-12 | AaBbDd | 142 -1 | 142 -1 | 142 -1 |
| Thatcher/239-1F1-52-13 | aaBBDd | 142 -1 |  | 142 -1 |
| Thatcher/239-1F1-52-14 | AABbDD |  | 142 -1 |  |
| Thatcher/239-1F1-52-15 | AabbDd | 142 -1 | 142 -1 | 142 -1 |
| Thatcher/239-1F1-52-19 | AABbDd |  | 142 -1 | 142 -1 |
| Thatcher/239-1F1-52-2 | AaBbDD | 142 -1 | 142 -1 |  |
| Thatcher/239-1F1-52-20 | AABbDd |  | 142 -1 | 142 -1 |
| Thatcher/239-1F1-52-21 | AABBDD |  |  |  |
| Thatcher/239-1F1-52-22 | AaBbDd | 142 -1 | 142 -1 | 142 -1 |
| Thatcher/239-1F1-52-23 | AaBbDd | 142 -1 | 142 -1 | 142 -1 |
| Thatcher/239-1F1-52-26 | AabbDd | 142 -1 | 142 -1 | 142 -1 |
| Thatcher/239-1F1-52-27 | Aabbdd | 142 -1 | 142 -1 | 142 -1 |
| Thatcher/239-1F1-52-28 | AabbDd | 142 -1 | 142 -1 | 142 -1 |
| Thatcher/239-1F1-52-30 | AaBbdd | 142 -1 | 142 -1 | 142 -1 |
| Thatcher/239-1F1-52-31 | AabbDd | 142 -1 | 142 -1 | 142 -1 |
| Thatcher/239-1F1-52-32 | aaBbDd | 142 -1 | 142 -1 | 142 -1 |
| Thatcher/239-1F1-52-33 | AaBbDD | 142 -1 | 142 -1 |  |
| Thatcher/239-1F1-52-34 | aaBbDD | 142 -1 | 142 -1 |  |
| Thatcher/239-1F1-52-35 | AaBbDd | 142 -1 | 142 -1 | 142 -1 |
| Thatcher/239-1F1-52-36 | AabbDD | 142 -1 | 142 -1 |  |
| Thatcher/239-1F1-52-38 | AaBbDD | 142 -1 | 142 -1 |  |
| Thatcher/239-1F1-52-39 | aabbDD | 142 -1 | 142 -1 |  |
| Thatcher/239-1F1-52-4 | AabbDd | 142 -1 | 142 -1 | 142 -1 |
| Thatcher/239-1F1-52-40 | AaBbDd | 142 -1 | 142 -1 | 142 -1 |
| Thatcher/239-1F1-52-41 | aaBbDd | 142 -1 | 142 -1 | 142 -1 |
| Thatcher/239-1F1-52-42 | AaBbdd | 142 -1 | 142 -1 | 142 -1 |
| Thatcher/239-1F1-52-43 | aabbDd | 142 -1 | 142 -1 | 142 -1 |
| Thatcher/239-1F1-52-44 | AaBbDd | 142 -1 | 142 -1 | 142 -1 |
| Thatcher/239-1F1-52-47 | aaBbDD | 142 -1 | 142 -1 |  |
| Thatcher/239-1F1-52-48 | AabbDD | 142 -1 | 142 -1 |  |
| Thatcher/239-1F1-52-49 | AaBbdd | 142 -1 | 142 -1 | 142 -1 |
| Thatcher/239-1F1-52-5 | AAbbdd |  | 142 -1 | 142 -1 |
| Thatcher/239-1F1-52-50 | AAbbDD |  | 142 -1 |  |
| Thatcher/239-1F1-52-51 | AABbdd |  | 142 -1 | 142 -1 |
| Thatcher/239-1F1-52-54 | AaBbDd | 142 -1 | 142 -1 | 142 -1 |
| Thatcher/239-1F1-52-55 | AAbbDd |  | 142 -1 | 142 -1 |
| Thatcher/239-1F1-52-56 | AAbbDd |  | 142 -1 | 142 -1 |
| Thatcher/239-1F1-52-57 | AAbbDd |  | 142 -1 | 142 -1 |
| Thatcher/239-1F1-52-58 | AabbDd | 142 -1 | 142 -1 | 142 -1 |
| Thatcher/239-1F1-52-59 | Aabbdd | 142 -1 | 142 -1 | 142 -1 |
| Thatcher/239-1F1-52-6 | AAbbdd |  | 142 -1 | 142 -1 |
| Thatcher/239-1F1-52-60 | AaBBDd | 142 -1 |  | 142 -1 |
| Thatcher/239-1F1-52-61 | AabbDD | 142 -1 | 142 -1 |  |
| Thatcher/239-1F1-52-62 | AAbbdd |  | 142 -1 | 142 -1 |
| Thatcher/239-1F1-52-7 | AaBbDD | 142 -1 | 142 -1 |  |
| Thatcher/239-1F1-52-8 | AABbDD |  | 142 -1 |  |
| Thatcher/239-1F1-6-1 | AabbDd | 142 -1 | 142 -1 | 142 -1 |
| Thatcher/239-1F1-6-10 | AABbDD |  | 142 -1 |  |
| Thatcher/239-1F1-6-11 | Aabbdd | 142 -1 | 142 -1 | 142 -1 |
| Thatcher/239-1F1-6-12 | AaBbDD | 142 -1 | 142 -1 |  |
| Thatcher/239-1F1-6-13 | AABbDd |  | 142 -1 | 142 -1 |
| Thatcher/239-1F1-6-14 | AABbDd |  | 142 -1 | 142 -1 |
| Thatcher/239-1F1-6-15 | AaBbDd | 142 -1 | 142 -1 | 142 -1 |
| Thatcher/239-1F1-6-16 | Aabbdd | 142 -1 | 142 -1 | 142 -1 |
| Thatcher/239-1F1-6-17 | AaBBDD | 142 -1 |  |  |
| Thatcher/239-1F1-6-18 | AaBBDd | 142 -1 |  | 142 -1 |
| Thatcher/239-1F1-6-19 | aaBBdd | 142 -1 |  | 142 -1 |
| Thatcher/239-1F1-6-2 | AaBbdd | 142 -1 | 142 -1 | 142 -1 |
| Thatcher/239-1F1-6-20 | aabbDD | 142 -1 | 142 -1 |  |
| Thatcher/239-1F1-6-22 | AAbbDd |  | 142 -1 | 142 -1 |
| Thatcher/239-1F1-6-25 | aabbdd | 142 -1 | 142 -1 | 142 -1 |
| Thatcher/239-1F1-6-26 | AaBBDD | 142 -1 |  |  |
| Thatcher/239-1F1-6-27 | AabbDd | 142 -1 | 142 -1 | 142 -1 |
| Thatcher/239-1F1-6-28 | aaBbDD | 142 -1 | 142 -1 |  |
| Thatcher/239-1F1-6-30 | AaBbdd | 142 -1 | 142 -1 | 142 -1 |
| Thatcher/239-1F1-6-31 | AAbbDd |  | 142 -1 | 142 -1 |
| Thatcher/239-1F1-6-32 | AabbDd | 142 -1 | 142 -1 | 142 -1 |
| Thatcher/239-1F1-6-33 | aabbDd | 142 -1 | 142 -1 | 142 -1 |
| Thatcher/239-1F1-6-34 | AaBbDD | 142 -1 | 142 -1 |  |
| Thatcher/239-1F1-6-35 | AABbdd |  | 142 -1 | 142 -1 |
| Thatcher/239-1F1-6-36 | AABbDD |  | 142 -1 |  |
| Thatcher/239-1F1-6-37 | AaBbDD | 142 -1 | 142 -1 |  |
| Thatcher/239-1F1-6-38 | AaBbDD | 142 -1 | 142 -1 |  |
| Thatcher/239-1F1-6-39 | AaBbDd | 142 -1 | 142 -1 | 142 -1 |
| Thatcher/239-1F1-6-4 | AaBbDd | 142 -1 | 142 -1 | 142 -1 |
| Thatcher/239-1F1-6-40 | aabbDd | 142 -1 | 142 -1 | 142 -1 |
| Thatcher/239-1F1-6-41 | AABBdd |  |  | 142 -1 |
| Thatcher/239-1F1-6-42 | AaBbDD | 142 -1 | 142 -1 |  |
| Thatcher/239-1F1-6-44 | AaBBDd | 142 -1 |  | 142 -1 |
| Thatcher/239-1F1-6-45 | AABbdd |  | 142 -1 | 142 -1 |
| Thatcher/239-1F1-6-46 | AABbDd |  | 142 -1 | 142 -1 |
| Thatcher/239-1F1-6-47 | Aabbdd | 142 -1 | 142 -1 | 142 -1 |
| Thatcher/239-1F1-6-5 | AaBbDd | 142 -1 | 142 -1 | 142 -1 |
| Thatcher/239-1F1-6-6 | AAbbDD |  | 142 -1 |  |
| Thatcher/239-1F1-6-7 | AaBbDD | 142 -1 | 142 -1 |  |
| Thatcher/239-1F1-6-8 | AaBbDd | 142 -1 | 142 -1 | 142 -1 |
| Thatcher/239-1F1-6-9 | AaBBdd | 142 -1 |  | 142 -1 |
| Thatcher/239-1F1-8-12 | aaBBDd | 142 -1 |  | 142 -1 |
| Thatcher/239-1F1-8-2 | AaBBdd | 142 -1 |  | 142 -1 |
| Thatcher/239-1F1-8-33 | AaBBDD | 142 -1 |  |  |
| Thatcher/239-1F1-8-44 | aaBBdd | 142 -1 |  | 142 -1 |
| Thatcher/239-1F1-8-51 | AaBBDd | 142 -1 |  | 142 -1 |
| Thatcher/239-1F1-9-1 | AaBbDd | 142 -1 | 142 -1 | 142 -1 |
| Thatcher/239-1F1-9-10 | aabbDd | 142 -1 | 142 -1 | 142 -1 |
| Thatcher/239-1F1-9-13 | AaBbdd | 142 -1 | 142 -1 | 142 -1 |
| Thatcher/239-1F1-9-14 | AABbDd |  | 142 -1 | 142 -1 |
| Thatcher/239-1F1-9-15 | AaBbdd | 142 -1 | 142 -1 | 142 -1 |
| Thatcher/239-1F1-9-16 | AaBbDd | 142 -1 | 142 -1 | 142 -1 |
| Thatcher/239-1F1-9-17 | AabbDD | 142 -1 | 142 -1 |  |
| Thatcher/239-1F1-9-2 | AaBbdd | 142 -1 | 142 -1 | 142 -1 |
| Thatcher/239-1F1-9-20 | AaBBDd | 142 -1 |  | 142 -1 |
| Thatcher/239-1F1-9-21 | AABbDd |  | 142 -1 | 142 -1 |
| Thatcher/239-1F1-9-22 | AabbDd | 142 -1 | 142 -1 | 142 -1 |
| Thatcher/239-1F1-9-23 | AaBbDd | 142 -1 | 142 -1 | 142 -1 |
| Thatcher/239-1F1-9-24 | AaBbDD | 142 -1 | 142 -1 |  |
| Thatcher/239-1F1-9-25 | AabbDd | 142 -1 | 142 -1 | 142 -1 |
| Thatcher/239-1F1-9-26 | AAbbDd |  | 142 -1 | 142 -1 |
| Thatcher/239-1F1-9-27 | AabbDd | 142 -1 | 142 -1 | 142 -1 |
| Thatcher/239-1F1-9-28 | AaBBDd | 142 -1 |  | 142 -1 |
| Thatcher/239-1F1-9-29 | AABbDD |  | 142 -1 |  |
| Thatcher/239-1F1-9-30 | AabbDd | 142 -1 | 142 -1 | 142 -1 |
| Thatcher/239-1F1-9-31 | AaBbDD | 142 -1 | 142 -1 |  |
| Thatcher/239-1F1-9-32 | aaBbDd | 142 -1 | 142 -1 | 142 -1 |
| Thatcher/239-1F1-9-33 | AaBbDd | 142 -1 | 142 -1 | 142 -1 |
| Thatcher/239-1F1-9-34 | AaBBDd | 142 -1 |  | 142 -1 |
| Thatcher/239-1F1-9-35 | AaBbdd | 142 -1 | 142 -1 | 142 -1 |
| Thatcher/239-1F1-9-36 | aaBbDD | 142 -1 | 142 -1 |  |
| Thatcher/239-1F1-9-37 | aaBBDD | 142 -1 |  |  |
| Thatcher/239-1F1-9-38 | AAbbDd |  | 142 -1 | 142 -1 |
| Thatcher/239-1F1-9-39 | AabbDd | 142 -1 | 142 -1 | 142 -1 |
| Thatcher/239-1F1-9-4 | AaBBDd | 142 -1 |  | 142 -1 |
| Thatcher/239-1F1-9-40 | AabbDd | 142 -1 | 142 -1 | 142 -1 |
| Thatcher/239-1F1-9-41 | AABbdd |  | 142 -1 | 142 -1 |
| Thatcher/239-1F1-9-42 | Aabbdd | 142 -1 | 142 -1 | 142 -1 |
| Thatcher/239-1F1-9-43 | AaBbDd | 142 -1 | 142 -1 | 142 -1 |
| Thatcher/239-1F1-9-44 | aaBBDd | 142 -1 |  | 142 -1 |
| Thatcher/239-1F1-9-45 | AaBbDd | 142 -1 | 142 -1 | 142 -1 |
| Thatcher/239-1F1-9-46 | aaBbDd | 142 -1 | 142 -1 | 142 -1 |
| Thatcher/239-1F1-9-47 | AAbbDd |  | 142 -1 | 142 -1 |
| Thatcher/239-1F1-9-48 | AAbbDD |  | 142 -1 |  |
| Thatcher/239-1F1-9-49 | AABbDd |  | 142 -1 | 142 -1 |
| Thatcher/239-1F1-9-5 | aaBBdd | 142 -1 |  | 142 -1 |
| Thatcher/239-1F1-9-50 | Aabbdd | 142 -1 | 142 -1 | 142 -1 |
| Thatcher/239-1F1-9-51 | AabbDD | 142 -1 | 142 -1 |  |
| Thatcher/239-1F1-9-52 | AaBbDD | 142 -1 | 142 -1 |  |
| Thatcher/239-1F1-9-53 | AAbbDd |  | 142 -1 | 142 -1 |
| Thatcher/239-1F1-9-54 | AaBBDd | 142 -1 |  | 142 -1 |
| Thatcher/239-1F1-9-55 | AaBBDd | 142 -1 |  | 142 -1 |
| Thatcher/239-1F1-9-56 | AabbDd | 142 -1 | 142 -1 | 142 -1 |
| Thatcher/239-1F1-9-58 | AAbbDd |  | 142 -1 | 142 -1 |
| Thatcher/239-1F1-9-59 | aabbDd | 142 -1 | 142 -1 | 142 -1 |
| Thatcher/239-1F1-9-6 | AaBbdd | 142 -1 | 142 -1 | 142 -1 |
| Thatcher/239-1F1-9-60 | AabbDd | 142 -1 | 142 -1 | 142 -1 |
| Thatcher/239-1F1-9-61 | aaBbDd | 142 -1 | 142 -1 | 142 -1 |
| Thatcher/239-1F1-9-62 | AABbdd |  | 142 -1 | 142 -1 |
| Thatcher/239-1F1-9-63 | AaBbDd | 142 -1 | 142 -1 | 142 -1 |
| Thatcher/239-1F1-9-64 | AABbDd |  | 142 -1 | 142 -1 |
| Thatcher/239-1F1-9-65 | AaBbdd | 142 -1 | 142 -1 | 142 -1 |
| Thatcher/239-1F1-9-66 | AabbDD | 142 -1 | 142 -1 |  |
| Thatcher/239-1F1-9-67 | AabbDd | 142 -1 | 142 -1 | 142 -1 |
| Thatcher/239-1F1-9-69 | AaBbdd | 142 -1 | 142 -1 | 142 -1 |
| Thatcher/239-1F1-9-70 | aaBbDd | 142 -1 | 142 -1 | 142 -1 |
| Thatcher/239-1F1-9-71 | aabbDd | 142 -1 | 142 -1 | 142 -1 |
| Thatcher/239-1F1-9-72 | AaBbDd | 142 -1 | 142 -1 | 142 -1 |
| Thatcher/239-1F1-9-73 | aabbDD | 142 -1 | 142 -1 |  |
| Thatcher/239-1F1-9-74 | AaBBDd | 142 -1 |  | 142 -1 |
| Thatcher/239-1F1-9-75 | AaBbdd | 142 -1 | 142 -1 | 142 -1 |
| Thatcher/239-1F1-9-8 | aaBBDD | 142 -1 |  |  |
| Thatcher/239-1F1-9-9 | AaBbdd | 142 -1 | 142 -1 | 142 -1 |

Note: The A/a, B/b, and D/d in the genotype represent the A, B, and D genome homoeologue respectively. The lower and upper case represent mutant and wild type alleles respectively. The mutations of each mutated allele are shown in the right three columns. The nucleotide “A” in the start codon is assigned as position 1. The start position of each mutation is shown on the left, and it is followed by the deletion or insertion of different number nucleotides. The deletion and insertion is shown as “-” and “+” respectively.

**Table S5. The EMS mutagenesis selection primers and Kaspar marker primers for Paragon population genotyping.**

| **Objective** | **Amplified region** | **Primer Name** | **Primer Sequence (5'-3')** | **Amplicon size (bp)** |
| --- | --- | --- | --- | --- |
| TILLING-*TaGW2-B* | Exons 2-6 | JB2_GW2_B_F2 | AACTCGTTAATAAGATTCCGCT | 1091 |
|  |  | JB7_GW2_B_R4 | CTTTTAGAGTAGCTGTCATCTGA |  |
| KASPar-*TaGW2-A* | G2373A SNP | *TaGW2_A_WT_FAM* | *GAAGGTGACCAAGTTCATGCTGCTTCAATGACTTTCTGTTCTTCc* | 121 |
|  |  | *TaGW2_A_M_HEX* | *GAAGGTCGGAGTCAACGGATTGCTTCAATGACTTTCTGTTCTTCt* |  |
|  |  | *TaGW2_A_C* | *AGAGCAATTTGTAAGTCTTATTCC* |  |
| KASPar-*TaGW2-B* | C2504T SNP | *TaGW2_B_WT_FAM* | *GAAGGTGACCAAGTTCATGCTCTCCAACAACAGAAGTGGAGTATc* | 123 |
|  |  | *TaGW2_B_M_HEX* | *GAAGGTCGGAGTCAACGGATTCTCCAACAACAGAAGTGGAGTATt* |  |
|  |  | *TaGW2_B_C* | *GTAAGTTATCAGATTAAGCTACAGG* |  |
| KASPar-*TaGW2-D* | G7139A SNP | *TaGW2_D_WT_FAM* | *GAAGGTGACCAAGTTCATGCTCATGATGGTTATGGAAGCGATTTg* | 145 |
|  |  | *TaGW2_D_M_HEX* | *GAAGGTCGGAGTCAACGGATTCATGATGGTTATGGAAGCGATTTa* |  |
|  |  | *TaGW2_D_C* | *GAAAACAATTTGATCCAACAAGTCA* |  |

Note: The black letters are target specific primers, the blue and red letters are common tails for FAM and HEX fluorescent signal respectively.

**Table S6. The *TaGW2* gene A, B, and D homoeologue specific primers for realtime PCR.**

| Name | Sequence |
| --- | --- |
| GW2A_RT_F | AAGCATGGGTGCTGCGGAA |
| GW2A_RT_R | GTCAGCAAAAGGCAACGGTA |
| GW2B_RT_F | AACGCCACCGTTGCCTGTTA |
| GW2B_RT_R | GGCAGGAAACGTCACAATCATA |
| GW2D_RT_F | AAGCATGGGTGCTGTGGAG |
| GW2D_RT_R | GCAAAAGGCAACGGTGGCA |
| TaActin-F | ACCTTCAGTTGCCCAGCAAT |
| TaActin-R | CAGAGTCGAGCACAATACCAGTTG |

**Table S7. Analysis of the relative expression levels of *TaGW2* homoeologues from tissues of cultivar Azhurnaya at various developmental stages.**

| **Tissue** | **Genome A** | | **Genome B** | | **Genome D** | |
| --- | --- | --- | --- | --- | --- | --- |
|  | **Expression Level** | **Standard Deviation** | **Expression Level** | **Standard Deviation** | **Expression Level** | **Standard Deviation** |
| First leaf sheath - Tillering stage | 2.95 | 0.14 | 5.44 | 1.35 | 3.21 | 0.6 |
| Internode #2 - Milk grain stage | 4.29 | 0.79 | 5.69 | 1.01 | 3.63 | 0.78 |
| Shoot apical meristem - Seedling stage | 3.65 | 0.17 | 5.3 | 0.42 | 4 | 0.07 |
| Grain - Milk grain stage | 4.29 | 1.04 | 4.53 | 0.7 | 3.86 | 0.32 |
| First leaf blade - Seedling stage | 1.7 | 0.44 | 2.31 | 1.07 | 1.69 | 0.69 |
| Flag leaf blade - Full boot | 2.44 | 0.11 | 2.99 | 0.26 | 2.28 | 0.15 |
| Awn - 50 percent spike | 3.9 | 0.58 | 6.19 | 0.47 | 4.74 | 0.26 |
| flag leaf blade night (-0.25h) 06:45 | 4.15 | 0.04 | 6.12 | 0.39 | 3.46 | 0.36 |
| Shoot axis - Flag leaf stage | 4.59 | 0.33 | 6.2 | 0.58 | 4.45 | 0.69 |
| Fifth leaf blade - Flag leaf stage | 2.28 | 0.8 | 2.86 | 1.23 | 2.45 | 0.63 |
| Third leaf sheath - Three leaf stage | 4.04 | 0.44 | 5.59 | 0.48 | 3.49 | 0.54 |
| Internode #2 - Ear emergence | 3.63 | 0.44 | 4.61 | 0.18 | 3.3 | 0.55 |
| Anther | 2.01 | 0.46 | 3.58 | 0.67 | 3.38 | 0.48 |
| Spike | 6.52 | 0.23 | 8.41 | 0.39 | 6.65 | 0.6 |
| Coleoptile | 4.43 | 1.18 | 5.96 | 1.2 | 5.34 | 0.77 |
| Stigma and Ovary | 6.51 | 0.39 | 8.91 | 1.74 | 6.38 | 0.26 |
| Roots - Flag leaf stage | 4.54 | 0.67 | 7.82 | 0.7 | 5.49 | 0.7 |
| Fifth leaf sheath - Flag leaf stage | 2.86 | 0.33 | 3.76 | 0.5 | 2.86 | 0.28 |
| Root apical meristem - Three leaf stage | 5.01 | 0.36 | 6.73 | 0.54 | 5.84 | 0.7 |
| Flag leaf sheath - Ear emergence | 3.13 | 0.67 | 3.48 | 0.4 | 3.12 | 0.17 |
| Roots - Three leaf stage | 5.79 | 0.74 | 8.43 | 1.73 | 5.88 | 1.59 |
| Flag leaf sheath - 50 percent spike | 3.07 | 0.53 | 4.26 | 0.35 | 3.51 | 0.27 |
| Radicle - Seedling stage | 4.55 | 0.42 | 6.35 | 1.05 | 5.6 | 0.62 |
| Roots - 50 percent spike | 4 | 0.91 | 6.67 | 2.11 | 4.47 | 1.14 |
| Third leaf blade - Three leaf stage | 1.63 | 0.49 | 1.93 | 0.57 | 1.87 | 0.47 |
| Spikelets - 50 percent spike | 4.28 | 0.61 | 5.49 | 0.95 | 4.4 | 0.3 |
| Root apical meristem - Tillering stage | 4.19 | 0.42 | 5.85 | 0.9 | 4.08 | 0.86 |
| Grain - Ripening stage | 13.04 | 4.34 | 20.51 | 4.23 | 16.05 | 5.84 |
| Awns - Ear emergence | 2.6 | 0.8 | 3.19 | 0.88 | 3.09 | 0.91 |
| Glumes | 4.36 | 0.76 | 5.73 | 0.04 | 4.61 | 0.65 |
| Glumes - Ear emergence | 4.35 | 0.34 | 4.75 | 0.8 | 4.2 | 0.19 |
| Leaf ligule | 3.28 | 0.03 | 3.99 | 0.68 | 4.12 | 0.81 |
| Flag leaf blade - 50 percent spike | 3.54 | 0.22 | 4.16 | 0.43 | 2.74 | 0.15 |
| Internode #2 - 50 percent spike | 4.54 | 0.43 | 7.07 | 0.6 | 4.04 | 0.52 |
| Fifth leaf sheath - Fifth leaf stage | 3.93 | 0.61 | 5.91 | 0.66 | 4.12 | 0.53 |
| fifth leaf blade night (-0.25h) 21:45 | 1.02 | 0.21 | 1.93 | 0.45 | 1.38 | 0.26 |
| Grain - Soft dough | 2.56 | 0.42 | 2.85 | 0.5 | 2.37 | 0.11 |
| Flag leaf blade (senescence) - Dough stage | 2.67 | 0.54 | 3.39 | 0.73 | 2.32 | 0.2 |
| Flag leaf blade night (-0.25h) 06:45 - Flag leaf stage | 1.75 | 1 | 2.09 | 1.1 | 1.65 | 0.56 |
| Flag leaf blade (senescence) - Ripening stage | 3.09 | 0.3 | 4.01 | 0.65 | 3.08 | 0.33 |
| First leaf blade - Tillering stage | 3.64 | 0.58 | 4.14 | 0.39 | 3.55 | 0.18 |
| Shoot apical meristem - Tillering stage | 3.73 | 0.35 | 5.76 | 0.45 | 3.42 | 0.42 |
| Shoot axis - First leaf stage | 3.07 | 1.03 | 4.14 | 1.14 | 3.24 | 1.41 |
| Roots - Seedling stage | 4.48 | 0.58 | 6.26 | 0.44 | 5.27 | 0.38 |
| Shoot axis - Milk grain stage | 4.75 | 0.06 | 6.09 | 0.41 | 4.16 | 0.15 |
| Fifth leaf blade - Fifth leaf stage | 1.52 | 0.79 | 2.27 | 1.08 | 1.36 | 0.7 |
| Flag leaf blade - Ear emergence | 2.83 | 0.26 | 3.16 | 0.04 | 2.46 | 0.18 |
| flag leaf blade night (+0.25h) 07:15 | 1.32 | 0.28 | 1.96 | 0.32 | 1.46 | 0.11 |
| Fifth leaf blade night (-0.25h) 21:45 | 1.99 | 0.26 | 3.39 | 0.37 | 1.95 | 0.08 |
| Shoot axis - Tillering stage | 3.58 | 0.26 | 5.58 | 0.71 | 3.85 | 0.11 |
| Stem axis - First leaf stage | 3.07 | 1.03 | 4.14 | 1.14 | 3.24 | 1.41 |
| Endosperm | 2.54 | 0.3 | 3.92 | 0.51 | 2.38 | 0.32 |
| Peduncle | 4.72 | 0.12 | 5.21 | 1.18 | 4.62 | 0.61 |
| Peduncle - 50 percent spike | 2.39 | 0.98 | 3.55 | 1.66 | 2.37 | 0.38 |
| Peduncle - Ear emergence | 2.56 | 0.59 | 2.79 | 0.3 | 2.3 | 0.24 |
| Flag leaf sheath - Full boot | 2.6 | 0.54 | 2.64 | 0.55 | 2.5 | 0.68 |
| Flag leaf blade - Flag leaf stage | 1.06 | 0.27 | 1.6 | 0.4 | 1.34 | 0.55 |
| Lemma | 4.77 | 0.4 | 5.42 | 0.77 | 3.71 | 0.56 |
| Lemma - Ear emergence | 4.62 | 1.07 | 4.82 | 0.95 | 3.98 | 0.5 |
| Awns - Milk grain stage | 4.44 | 0.22 | 4.49 | 0.47 | 4.36 | 0.09 |
| fifth leaf blade night (+0.25h) 22:15 | 1.52 | 0.19 | 1.85 | 0.33 | 1.52 | 0.19 |
| Flag leaf blade - Milk grain stage | 2.6 | 0.26 | 2.94 | 0.72 | 2.38 | 0.24 |
| Grain - Hard dough | 6.46 | 2.8 | 11.69 | 4.12 | 6.92 | 3.74 |
| Flag leaf sheath - Milk grain stage | 2.85 | 0.16 | 4.49 | 0.52 | 3.19 | 0.66 |
| Embryo proper | 11.35 | 0.47 | 21.48 | 3.25 | 13.03 | 1.77 |
| Fifth leaf blade (senescence) - Milk grain stage | 3.04 | 0.62 | 3.79 | 0.57 | 2.67 | 0.44 |
| Roots - Tillering stage | 4.7 | 0.48 | 6.75 | 0.67 | 4.32 | 0.24 |
| Shoot axis - Full boot | 4.44 | 0.21 | 7.26 | 0.91 | 3.81 | 0.01 |
| Fifth leaf blade - Ear emergence | 2.13 | 0.97 | 2.59 | 0.23 | 2.25 | 0.72 |
| First leaf sheath - Seedling stage | 2.71 | 0.88 | 3.5 | 1.19 | 3.24 | 0.91 |

Note: The expression values are shown as Transcripts Per Million (TPM) based on RNA-Seq data.
